# Commercial bioinoculants improve colonization but do not alter the arbuscular mycorrhizal fungal community of greenhouse-grown grapevine roots

**DOI:** 10.1186/s40793-025-00676-8

**Published:** 2025-01-31

**Authors:** Mariam P. Berdeja, Nicole K. Reynolds, Teresa Pawlowska, Justine E. Vanden Heuvel

**Affiliations:** 1 Horticulture Section, School of Integrative Plant Science, Cornell University, Ithaca, NY USA; 2 Present Address: Department of Plant and Soil Science, Texas Tech University, Lubbock, TX USA; 3 Plant Pathology and Plant Microbe-Biology Section, School of Integrative Plant Science, Cornell University, Ithaca, NY USA; 4 Present Address: Biological Sciences Department, California State Polytechnic University, Pomona, CA USA

**Keywords:** Arbuscular mycorrhizal fungi, Metabarcoding, Bioinoculants, Root traits, Biomass, Grapevine

## Abstract

**Background:**

Arbuscular mycorrhizal fungi (AMF) are beneficial root symbionts contributing to improved plant growth and development and resistance to abiotic and biotic stresses. Commercial bioinoculants containing AMF are widely considered as an alternative to agrochemicals in vineyards. However, their effects on grapevine plants grown in soil containing native communities of AMF are still poorly understood. In a greenhouse experiment, we evaluated the influence of five different bioinoculants on the composition of native AMF communities of young Cabernet Sauvignon vines grown in a non-sterile soil. Root colonization, leaf nitrogen concentration, plant biomass and root morphology were assessed, and AMF communities of inoculated and non-inoculated grapevine roots were profiled using high-throughput sequencing.

**Results:**

Contrary to our predictions, no differences in the microbiome of plants exposed to native AMF communities versus commercial AMF bioinoculants + native AMF communities were detected in roots. However, inoculation induced positive changes in root traits as well as increased AMF colonization, plant biomass, and leaf nitrogen. Most of these desirable functional traits were positively correlated with the relative abundance of operational taxonomic units identified as *Glomus*, *Rhizophagus* and *Claroideoglomus* genera.

**Conclusion:**

These results suggest synergistic interactions between commercial AMF bioinoculants and native AMF communities of roots to promote grapevine growth. Long-term studies with further genomics, metabolomics and physiological research are needed to provide a deeper understanding of the symbiotic interaction among grapevine roots, bioinoculants and natural AMF communities and their role to promote plant adaptation to current environmental concerns.

**Supplementary Information:**

The online version contains supplementary material available at 10.1186/s40793-025-00676-8.

## Background

Grapevine (*Vitis vinifera* L.) is a perennial crop well-adapted to different environmental conditions. However, projected climate change is expected to threaten grape production as well as grape berry quality and wine typicity, *i.e.* the degree to which a wine reflects its varietal origins [1, 2]. In addition, some viticultural practices, in particular the use of synthetic fertilizers and chemical pesticides, have a significant impact on soil, human, and environmental health and contribute to climate change [3]. The use of bioinoculants containing beneficial soil microorganisms has been proposed as an alternative to synthetic fertilizers and pesticides [3] and as a tool to regulate plant responses to different stresses associated with climate change [4].

Among beneficial microorganisms, arbuscular mycorrhizal fungi (AMF, Glomeromycotina) are recognized as key plant symbionts in sustainable agricultural ecosystems. AMF are obligate biotrophs, forming mutualistic symbiotic association with ~ 70% of land plants [5] and abundantly present in the soil of most ecosystems [6]. In this mutualistic association the host plant supplies carbohydrates and lipids to the fungus, which in return provides soil minerals and water to the plant [7]. Hence, AMF play a critical role in plant nutrition and health by improving soil quality and reducing the use of chemical fertilizers and pesticides [8, 9] under different environmental conditions. Given these ecological benefits, AMF have been harvested and applied as bioinoculants to improve nutrient use efficiency and crop yield [10, 11].

The AMF symbiosis has increasingly been shown to play an important role in viticulture resilience [12–14]. AMF inoculation can improve grapevine nutrition by increasing the availability and translocation of various nutrients, mainly phosphorus, nitrogen, potassium, calcium, and magnesium [14–16], maintain soil aggregate stability [12], and enhance resistance to various stresses including drought [12, 13], salinity [8], heavy metals [12, 16], viruses [17], and pathogens [18, 19]. Another key aspect of the AMF-host plant symbiosis is the effect on the root system [20]. For the plant, root system architecture is known to play a primary role in mineral nutrient acquisition [21]. Moreover, different aspects of root morphology and architecture are main drivers for AMF colonization and composition [22]. Recent research under natural conditions suggests that AMF inoculation may affect certain traits of grapevine root morphology [14] and grape berry primary and secondary metabolism [13, 23] in response to different environmental stresses. However, the effectiveness of AMF bioinoculants in promotion of plant growth and health have often been inconsistent and differ within fungal taxa and among plant hosts [11, 24]. Furthermore, the variable capacity of AMF to colonize different plant roots under different environmental conditions in greenhouse and field experiments has hindered their adoption by agronomical and perennial crop farmers [10, 11, 25].

In natural environments, plant root systems interact with and host multiple AMF species [24], which is associated with a diverse range of functional traits that allow AMF to colonize and benefit the host plant [26]. Previously, it has been proposed that functional traits and life history strategies of both AMF and host may lead to preferential partner selection in the plant and AMF symbiosis [26]. Consequently, AMF with contrasting growth and survival strategies have been classified as competitors (C), stress tolerators (S) and ruderals (R) in the CSR framework [26]. This framework was previously suggested to classify plant life-history strategies [27]. In this sense, some AMF species are more efficient at improving plant nutrient absorption, while others are better at enhancing resistance to different stresses [26, 28]. Therefore, the combination of multiple AMF species could better promote plant growth and plant ability to tolerate biotic and abiotic stresses than a single species [29, 30].

Despite the importance of AMF bioinoculants in sustainable agriculture, few studies have investigated the AMF composition of these commercial bioinoculants [31, 32] and the prevalence of AMF bioinoculant species in the inoculated roots [31, 33]. By performing restriction fragment length polymorphism (RFLP), Berruti et al. (2013) found that two single isolates, a *Rhizophagus* sp. (OTU1) and *Funneliformis mosseae* BEG12, were present in the roots of *Camellia japonica L*. (Theaceae, Theales) inoculated with mixed and single inoculum, respectively. Also, a recent study performing a molecular screening in eleven commercial inoculants found a contrasting mismatch between the AMF species composition indicated in the product labels and that found by sequencing [32]. However, it is unknown whether all the AMF species listed in mixed commercial bioinoculants colonize plant roots or if only few AMF species dominate the host colonization, and what effect competition with native species may have on these interactions.

Using *Vitis vinifera* Cabernet Sauvignon cultivated in non-sterile orchard soil and inoculated with five commercial bioinoculants containing different AMF species, this study addressed the following questions: (i) Do commercial bioinoculants increase root colonization and influence grapevine growth, leaf nutrient concentration and root morphology of inoculated plants when compared to non-inoculated plants? (ii) Does the AMF community of inoculated roots differ significantly from non-inoculated roots? (iii) To what extent do commercial bioinoculants alter the native AMF community diversity and composition of roots growing in non-sterile soil when compared to non-inoculated roots? Based on a previous study [14], we hypothesized that applying commercial bioinoculants to grapevine roots would result in greater root colonization, and improvement of overall growth, leaf nutrient concentration, and root architecture in grapevines. We also anticipated that not all the AMF species listed in the bioinoculants would colonize grapevine roots and the AMF community in roots will differ between inoculated and non-inoculated treatments.

## Material and methods

### Biological materials and experimental design

The experiment was conducted from June to December 2018, using two-year-old own-rooted *Vitis vinifera* L. cv. Cabernet Sauvignon. Plants were trained to two shoots and all lateral shoots were removed throughout the experiment. Dormant vines were planted in excavated (top 0-30 cm) apple orchard soil sourced from Cornell conventional Orchards, Ithaca, NY, containing native AMF species. To provide a more realistic scenario with a natural symbiotic community present in agricultural settings, we did not sterilize the orchard soil. The silt loam soil had a neutral pH and was low in available nitrogen (N), phosphorus (P) and potassium (K), while the concentration of boron (B), zinc (Zn) and manganese (Mn) were in adequate range (Supp. Table 1). The collected soil was sieved to 4 mm to remove any roots and plant material. The plants were grown in seven-liter pots filled with the sieved soil under controlled conditions with 16/8h light (high-pressure sodium lamps) and dark regime and day/night temperatures of 25 and 21 °C, respectively.

The experiment was laid out as a randomized complete block design with six treatments and four replicates each, giving a total of twenty-four experimental units (Supp. Figure 1). The treatments included non-inoculated (Control) treated with five autoclaved commercial bioinoculants and inoculated vines treated with one of five commercial bioinoculants, that were designated as product 1 (four AMF species), product 2 (nine AMF species), product 3 (nine AMF species), product 4 (nine AMF species) and product 5 (four AMF species). Products 2 and 5 contained only AMF species, while the other products included bacteria, ectomycorrhizal fungal species, and abiotic amendments (Supp. Table 2). All bioinoculants were applied directly into the root zone according to the manufacturer’s recommended rate of 14 g of granular bioinoculant per one vine for products 2, 4 and 5, and 14 g per two vines diluted in 1 gallon of water for products 1 and 3. To ensure adequate growth, 100 ml of a low-P fertilizer solution (15 N: 2.1 P: 12.4 K) was provided to all treatments weekly for the first six weeks and twice a week for the remaining 18 weeks of the experiment. Plants were watered two times per week. Twenty-four weeks after inoculation, vines were destructively harvested. Plants were divided into four organs: leaves/petioles for nutrient analysis, shoots, trunk, and roots for determination of biomass, as well as for root traits, mycorrhizal colonization, and molecular analysis.

### Plant phenotyping and sampling of roots

Leaf blades and petioles were combined for C and N analyses. All leaves were collected, washed with distilled water, dried with paper towel, and analyzed by the Cornell University Nutrient Analysis Laboratory to determine total C and N concentration via combustion analysis (Primacs; Skalar, Inc., Bufford, GA). The ratio of carbon-to-nitrogen (C: N) was acquired by dividing C by N. Shoots, trunks and roots were separated and washed in distilled water. Fresh weight (g FW per pot) was assessed and then samples were oven-dried at 60°C for 72 h until reaching a constant mass and weighed to record the dry weight (g DW per pot). The ratio of root to shoot (R: S) weight was obtained by dividing root dry weight by shoot dry weight.

Ten grams of roots were randomly sampled from four different quadrats of each root system, followed by careful removal of soil aggregates by manual shaking. Seven grams of each root sample was surface sterilized with 70% ethanol (v/v) for two minutes, followed by soaking in 1% hypochlorite sodium solution for 1 min, and careful rinsing with sterile milli-Q water three times to remove chemical residues. These surface sterilized fine roots were stored at -80°C for further DNA extraction. The other three grams of roots were carefully rinsed three times with distilled water and stored in 15% ethanol (v/v) at 4°C for fine root morphology analysis and AMF quantification. Root order was determined according to the method of Guo et al. (2008) [34] and McCormack et al. (2015) [35]. Roots were separated into absorptive (first- and second-order) and transportive (third-order and higher) fine roots. Only absorptive fine roots were scanned for image analysis (WinRhizo; Regent Instruments Inc., Québec City, QC, Canada). Root diameter (RD; mm) and total root length (RL; cm) of each sample were measured. The roots were then oven-dried at 60°C for 48 h and weighed to calculate root length density (RLD; cm cm^−3^ soil) and specific root length (SRL; m g^−1^ root), following the formulas:

1 $$RLD\left( {cm cm} \right)^{ - 3} = \frac{RL}{V}$$

2 $${\text{SRL}}\left( {{\text{m g}}^{ - 1} } \right) = \frac{{{\text{RL}}}}{RM}$$

where RL is the root length, V is the soil volume and RM is the root dry weight.

Following measurement of root morphology, fine root samples were rehydrated and stored in 70% (v/v), ethanol. To visualize mycorrhizal colonization, rehydrated fine roots were cut into 2-cm sections, cleared and stained according to Koske and Gemma (1989) [36] with some modifications: fragmented fine roots were cleared in 10% (w/v) KOH (90°C, 20 min), bleached with alkaline H_2_O_2_ solution (30 min), acidified with 1% (v/v) HCL (30 min), stained in 0.05% trypan blue (90°C, 25 min) in acidic glycerol solution and de-staining in 50% (v/v) glycerol (72h). The magnified intersections method [37] was used to determine the proportion of total root length colonized (RLC) by AMF. Briefly, this method assessed the presence and absence of fungal arbuscules, vesicles and hyphae in 100 intersects (per root sample), observed along the root length.

### Sample DNA extraction, amplification, and sequencing

DNA extractions of 24 frozen Cabernet Sauvignon root samples inoculated and not inoculated with commercial bioinoculants were performed using DNeasy® Plant Mini kit (Qiagen, USA) protocol, with the exception that polyvinylpyrrolidone (PVP40, 1% w/v final concentration) was added to the initial extraction buffer (AP1) to precipitate phenolic compounds. To monitor potential background contamination among samples, control extractions and PCRs were also included.

After DNA extraction, all samples were prepared for Illumina sequencing through PCR amplification, First the SSU (18S) and internal transcribed spacer (ITS2) rRNA gene regions were amplified separately for each sample. The SSU (18) gene was amplified using AMF-specific primers WANDA [38] and AML2 [39], while the ITS2 region was amplified using a mixture of fungal-specific forward primers ITS7 and ITS7o [40, 41] and the general eukaryotic primer ITS4 [42]. AMF specific primers were included alongside general fungal primers because ITS2 primers may not provide a comprehensive characterization of AMF communities due to poor AMF amplification [43]. Each primer was flanked by an Illumina Nextera adapter sequence (5′‐ TCGTCGGCAGCGTCAGATGTGTATAAGAGACAG‐forward_primer‐3′, 5′‐ GTCTCGTGGGCTCGGAGATGTGTATAAGAGACAG‐reverse_primer‐3′; Illumina, San Diego, California, USA).

PCR reactions were performed in 50 μL reaction volumes containing 1 ng of DNA extract as template, 0.5 µM of each primer, 10 µL 5X Phusion HF buffer, 200 µM each dNTPs, and 0.02 U/µL Phusion High-Fidelity DNA polymerase (Thermo Scientific, USA). Each reaction was carried out in triplicate in a Biorad MyCycler™ Thermal Cycler (Biorad, USA) under the following conditions: an initial denaturation at 98 °C for 3 min followed by 35 cycles at 98 °C for 10 s, 54 °C (for SSU) or 57 °C (for ITS) for 30 s, and 72 °C for 25 s, with a final elongation at 72 °C for 10 min. Negative and positive PCR controls were included in all reactions. To confirm the presence of target amplicons, all reactions were analyzed using1.5% agarose gel electrophoresis. Amplicons generated during this first PCR step were diluted (to a final concentration of 2–5 ng/µl) and used as templates in a second PCR step to add the barcodes (i.e., indexing reactions with Illumina Nextera barcodes). PCR2 amplicons were purified using AMPure XP beads (Beckman Coulter Genomics, USA), quantified by Qubit 2.0 fluorometer (Invitrogen, USA) and pooled in equimolar concentration prior to sequencing. Sequencing was performed at the Cornell Institute of Biotechnology (BRC, https://www.biotech.cornell.edu/) using 2 × 300 bp paired-end (v3) run on an Illumina MiSeq platform (Illumina Inc, CA, USA).

### Bioinformatic and statistical analysis

All Illumina data were processed using the AMPtk bioinformatics pipeline v1.5.5 [44]. For 18S rRNA data, the DADA2 wrapper [45] was used for denoising and amplicon sequence variant identification, followed by clustering using vsearch v2.22.1 [46] at 99% similarity for 18S OTU generation. A custom database comprising reference 18S rRNA sequences downloaded from GenBank as well as virtual taxa (VT) from the MaarjAM database [47] was made and installed in AMPtk to assign taxonomy. The final database consisted of 1,856 dereplicated sequences and is available at OSF (https://osf.io/kp65c/), 10.17605/OSF.IO/KP65C. For ITS rRNA data, sequences were clustered at 97% using UNOISE3 [48] and the UNITE database (v8.3 2021–11-25) [49] was used for taxonomy assignment. For both 18S and ITS, up to two nucleotide mismatches were allowed in each primer, a maximum of one expected error was allowed during demultiplexing and quality filtering and reference-based chimera filtering was used during clustering of ITS2 amplicons. OTUs detected in the negative controls and those identified as non-AMF (18S rRNA) or non-fungal (ITS rRNA) were removed from the datasets prior to further analyses. The AMF community detected by ITS rRNA was also analyzed separately from the total fungal community. All sequencing data were uploaded to the NCBI Sequence Read Archive accession number PRJNA1136973.

Calculation of diversity measures and assessment of community differences were performed with R statistical interface (v4.2.1) [50]. The phyloseq package [51] was used to create relative abundance bar graphs by bioinoculant product type and boxplots of alpha diversity. Alpha diversity was calculated for the 18S data, and data were rarefied to 25,000 sequencing depth prior to diversity analysis (Supp. Figure 2). To analyze beta diversity, OTU tables were first transformed into presence/absence format, and Simpson dissimilarity (βsim) matrices were calculated with the betadiver function in the vegan package [52] using the “w” method [53]. Community level differences were assessed with the metaMDS function in vegan and plotted using ggplot2 [54] and visualized using nonmetric multidimensional scaling (NMDS) ordinations. To test for significant dispersion (within-group variation) among bioinoculant product type, an ANOVA analysis of the βsim distance matrices was conducted (betadisper function in vegan R package) using centroid differences (type = “centroid”). Statistical tests to compare the effect of product on the fungal community were conducted with permutational multivariate analysis of variance (PERMANOVA) [55] of the βsim distance matrices [56] using the adonis2 function in the vegan R package.

Upset plots to visualize intersections of data between bioinoculant product type were created with the Complex Upset R package [57, 58]. To test for differences in plant traits by product type, ANOVA analyses followed by Tukey tests were conducted and plotted as boxplots using ggplot2 with the cowplot add-on. Finally, to investigate which OTUs may be linked to specific plant traits, Spearman correlation matrices with Holm p-value adjustments were calculated with the psych R package [59]. Correlation tests were also conducted for all OTUs/plant traits, and only those with a p-value < 0.05 were retained for the analysis. Data visualization was performed using ggcorrplot [60]. All R code is available at OSF (https://osf.io/kp65c/), 10.17605/OSF.IO/KP65C.

## Results

### AMF root colonization and grapevine growth performance

**Fig. 1:**
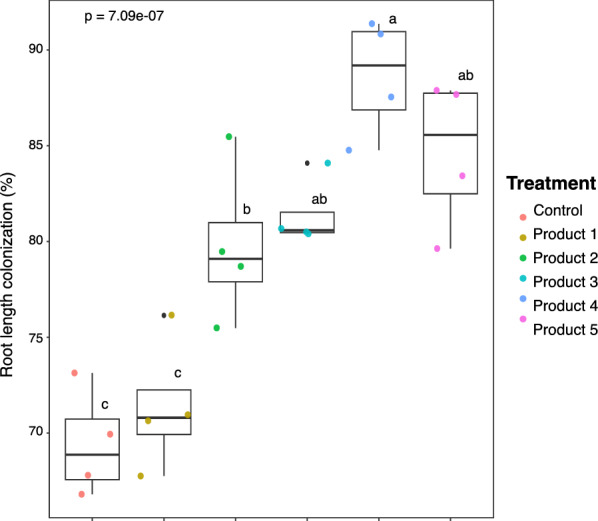
Total percent of the root length colonized by arbuscular mycorrhizal fungi (AMF) in fine roots of *Vitis vinifera* cv. Cabernet Sauvignon (*n* = 4), inoculated and non-inoculated (Control), with five bioinoculants. The boxplots show the first and third quartile ranges, with the line in the box representing the median. The whiskers extend from the first and third quartiles to values that are not within 1.5 × interquartile range from both directions. Data beyond the whiskers are presented as individual circles. Letters indicate differences in AMF colonization among inoculum treatments detected using Tukey’s honest significant difference post-hoc test derived from the linear model analysis of variance at α = 0.05 and ANOVA p-values shown

Plants treated with commercial bioinoculants increased the percentages of total mycorrhizal colonization (RLC) and mycorrhizal structures associated with roots colonized (vesicles, arbuscules, and hyphae) compared with control plants, except for hyphae and RLC in plants inoculated with product 1. Plants inoculated with products 4, 5, 3 and 2 increased by 19%, 15%,12% and 10% respectively (Fig. 1). Similarly, plants inoculated with products 4, 5, 3 and 2 displayed higher percentages of arbuscules, vesicles and hyphae colonization when compared to control plants (Supp. Figure 3).

Regardless of the treatment, N concentration in leaf blades and petioles was improved when compared with control plants. Plants inoculated with products 4, 5, and 3 respectively showed a higher N increase compared to plants inoculated with products 1 and 2 (Supp. Figure 4A). For all treatments, no significant effect of bioinoculants on C concentration was observed (Supp. Figure 4B). The increase in N but not in C concentration significantly decreased C: N ratios for inoculated plants (Supp. Figure 4C). Vines inoculated with products 4, 5, and 3, respectively showed a greater reduction of C: N ratios when compared to control plants.

Commercial bioinoculants significantly affected plant dry biomass (g) when compared to control plants (Supp. Figure 5). The effect on shoot and trunk was greater for plants inoculated with products 4, 5, 3 and 2, respectively, except for products 1 and 2 on shoot and product 1 on trunk biomass (Supp. Figure 5A and 5B). For instance, root biomass increased 75%, 65%, 43% and 49% in plants inoculated with products 4, 5, 3 and 2, respectively when comparing with control plants (Supp. Figure 5C**)**. No significant effect of bioinoculants on the R: S ratio was observed (Supp. Figure 5D).

### AMF root colonization effects on root morphology

**Fig. 2:**
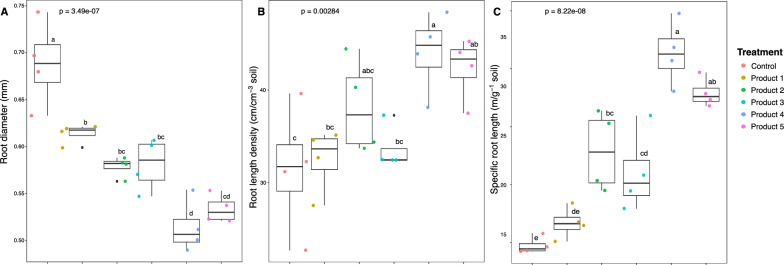
Root morphological traits; root diameter (**A**), root length density (**B**) and specific root length (**C**) of *Vitis vinifera* cv. Cabernet Sauvignon (n = 4), inoculated and non-inoculated (Control), with five bioinoculants. The boxplots show the first and third quartile ranges, with the line in the box representing the median. The whiskers extend from the first and third quartiles to values that are not within 1.5 × interquartile range from both directions. Data beyond the whiskers are presented as individual circles. Letters indicate differences in root measurements among inoculum treatments detected using Tukey’s honest significant difference post-hoc test derived from the linear model analysis of variance at α = 0.05 and ANOVA p-values shown

Fine root morphology was altered by inoculation (Fig. 2), except for RL (Supp. Figure 4D). Plants inoculated with products 4, 5, and 2 had the greater decrease in RD with 25%, 22% and 16%, respectively when compared to control plants (Fig. 2A). The greatest RLD was observed in plants inoculated with products 4, 5, and 2, by up to 40, 35 and 22%, respectively when contrasted with control plants (Fig. 2B). SRL was increased by 16.6 mg^−1^, 13.1 mg^−1^ and 8.1 mg^−1^ for plants inoculated with products 4, 5, and 2, respectively (Fig. 2C).

### Amplicon community data

**Fig. 3:**
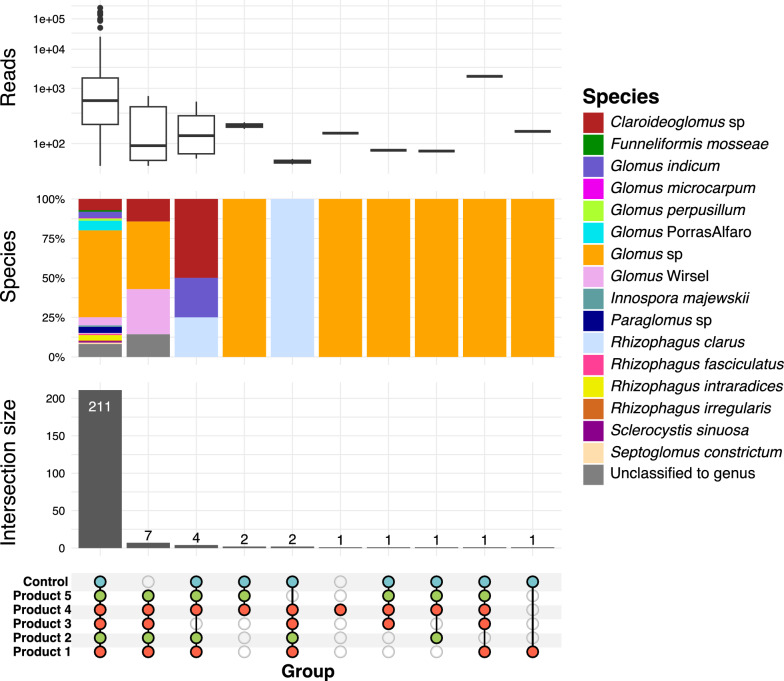
UpSet plot for *Vitis vinifera* cv. Cabernet Sauvignon inoculated and non-inoculated (Control), with five bioinoculants, displaying the total number of reads (log transformed), arbuscular mycorrhizal fungi (AMF) taxa composition and total number of shared or unique AMF OTUs according to the intersection matrix. Connected dots represent a certain intersection of OTUs among treatments. Numbers above vertical bars represent the number of AMF OTUs for each unique or overlapping combination found in the treatments marked by the colored dots

The ITS2 dataset had a total of 9,212,112 reads, with a range of 31,514 – 297,702 reads/sample, resulting in 1,003 OTUs prior to filtering. The 18S dataset had 7,893,325 total reads, with a range of 3,307 – 46,332 reads/sample, and 524 OTUs prior to filtering. The fungal community as recovered by ITS2 was largely dominated by Ascomycota and Basidiomycota, followed by Glomeromycetes class with 216 AMF OTUs (Supp. Figure 6). After filtering, the 18S community comprised 331 AMF OTUs, with the majority classified to various unidentified *Glomus* strains (Fig. 3). Significant within group dispersion by treatment was found for the AMF community in both datasets, but not in the overall ITS2 fungal community. However, no significant differences by treatment were detected in any of the fungal communities according to PERMANOVA analyses (Supp. Table 3). Upset plot results indicated that the majority of OTUs were shared across treatments, with only 7 OTUs detected in the product treatments but not in the controls (Fig. 3).

### AMF community richness, diversity, and identification

**Fig. 4:**
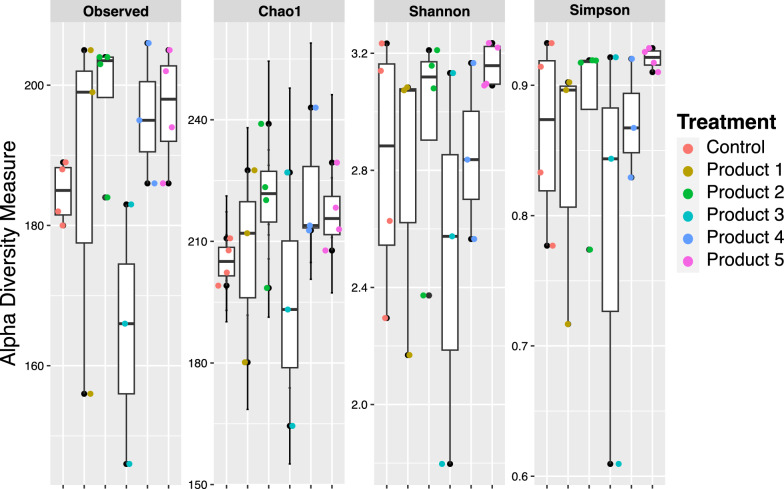
Boxplots comparison for Observed richness, Chao 1, Shannon, and Simpson diversity metrics of 18S rRNA-based arbuscular mycorrhizal fungal operational taxonomic units (OTUs) communities colonizing roots of *Vitis vinifera* cv. Cabernet Sauvignon inoculated and non-inoculated (Control), with five bioinoculants. The boxplots show the first and third quartile ranges, with the line in the box representing the median. The whiskers extend from the first and third quartiles to values that are not within 1.5 × interquartile range from both directions. Data beyond the whiskers are presented as individual circles

**Fig. 5:**
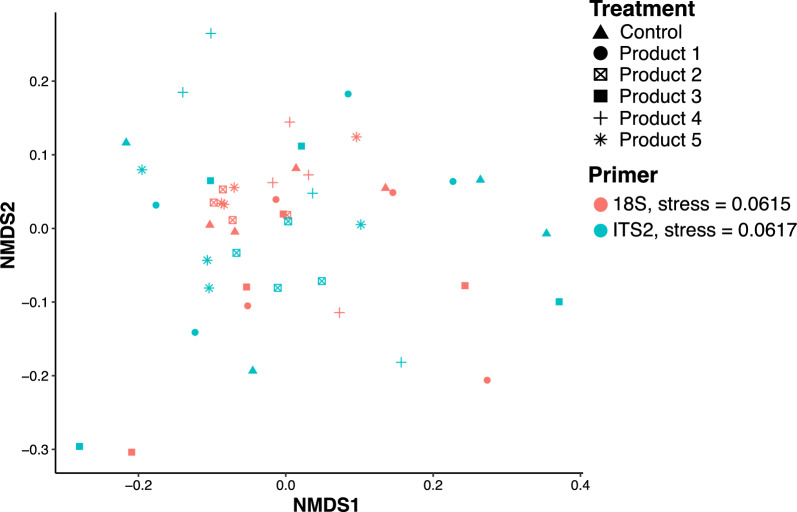
Non-metric multidimensional scaling (NMDS) ordination plot of arbuscular mycorrhizal fungal operational taxonomic units (OTUs) communities colonizing roots of *Vitis vinifera* cv. Cabernet Sauvignon inoculated and non-inoculated (Control), with five bioinoculants. Different colors represent communities from different primers sets (18S and ITS2 rRNA region), while shapes represent communities from different treatments

The diversity (Shannon and Simpson) and richness (Chao 1) indices of the AMF communities colonizing roots did not differ between inoculated and non-inoculated control plants (Fig. 4), indicating that AMF species from commercial bioinoculants had no significant effect on the diversity and richness of the AM fungal community found in the roots growing in excavated and non-sterile orchard soil. Non-metric multidimensional scaling (NMDS) ordination also did not vary in the mycorrhizal community composition found in the roots of treated and control plants (Fig. 5), showing that inoculated and non-inoculated control plants cultivated in the excavated and non-sterile orchard soil have similar AMF composition.

**Fig. 6:**
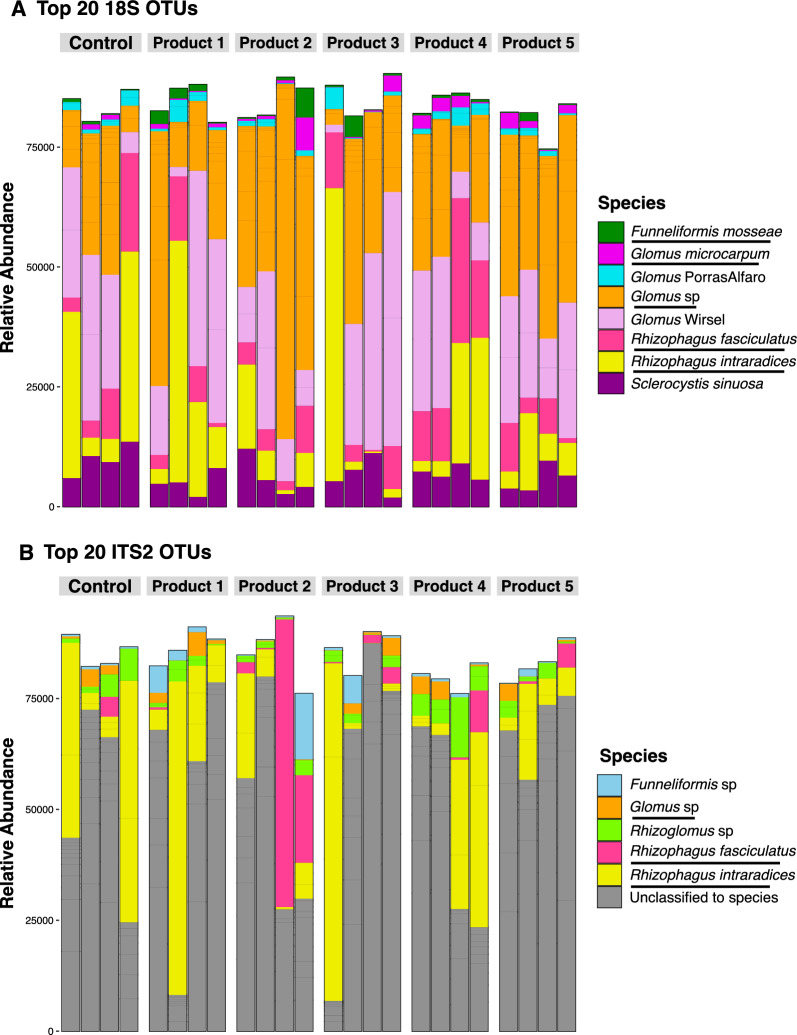
Taxonomic composition of the top 20 most abundant AMF operational taxonomic units (OTUs) for 18S (**A**) and ITS2 (**B**) rRNA gene regions, associated with roots of *Vitis vinifera* cv. Cabernet Sauvignon, inoculated and non-inoculated (Control) with five bioinoculants. Underlined names represent similar OTUs detected by both gene regions (less abundant OTUs not shown). *Glomus* Wirsel and *Glomus* PorrasAlfaro are based off the MaarjAM virtual taxa (VT) sequences, included in the reference 18S database installed in the AMPtk pipeline. Stacked bars represent relative abundance (percentage) and are colored by species identification. For full taxonomic diversity see Supp. Figures 6 and 7

Moreover, the relative abundance of AMF OTUs colonizing the roots did not differ among inoculated and non-inoculated control plants (Fig. 6, Supp. Figure 7). The most abundant genera identified in roots of inoculated and non-inoculated control plants by metabarcoding of 18S and ITS2 rRNA regions were *Glomus*, followed by *Rhizophagus*, and *Funneliformis* (Fig. 6), while *Claroideoglomus*, *Diversispora*, *Paraglomus*, and *Septoglomus* were less abundant (Supp. Figure 7). The AMF community compositions recovered by metabarcoding were compared with the commercial bioinoculants constituents. At the species level, only *Rhizophagus intraradices*, and *Funneliformis mosseae* were found in abundance in roots inoculated with commercial bioinoculants. According to the manufacturer, *Rhizophagus clarus* was not listed as being present in products 1 and 5*,* however, we found *Rhizophagus clarus* colonizing the roots of plants treated with products 1 and 5 (Supp. Figure 7). This result could potentially be attributed to the presence of the genus *Rhizophagus* in the excavated, non-sterile orchard soil used in this experiment.

### Relationship between AMF community and plant above and belowground metrics

**Fig. 7:**
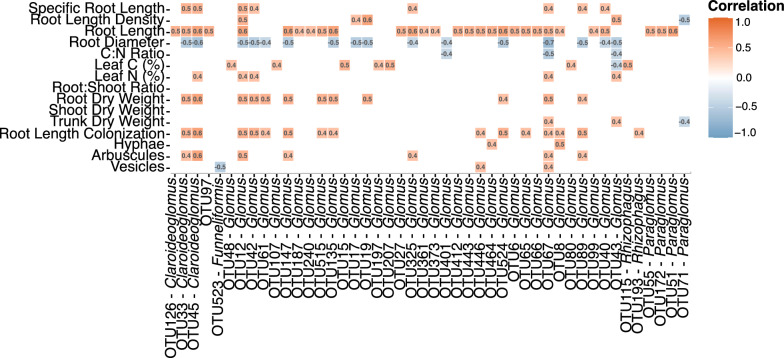
Spearman’s correlation between 46 key arbuscular mycorrhizal fungi 18S rRNA operational taxonomic units (OTUs) and physiological and morphological parameters of *Vitis vinifera* cv. Cabernet Sauvignon, inoculated and non-inoculated (Control), with five bioinoculants. The number in the boxes indicate Spearman’s rank correlation coefficient. Red boxes indicate positive correlation, and blue boxes indicate negative correlation

Spearman correlation co-efficient analysis was used to further explore the link between 46 key AMF OTUs (belonging to *Claroideoglomus*, *Funneliformis*, *Glomus*, *Paraglomus* and *Rhizophagus* genera) and their association with plant physiological and morphological parameters (Fig. 7). Fifteen of these OTUs were positively correlated to RLC. Arbuscule and hyphae structures displayed positive correlation with nine *Claroideoglomus* and *Glomus* OTUs. Vesicle structure was positively correlated with two *Glomus* OTUs, but negatively correlated to OTU 523, identified as *Funneliformis*. Twelve *Glomus* OTUs were positively correlated with root biomass. Twenty-nine OTUs identified as *Claroideoglomus*, *Glomus*, and *Paraglomus* were positively correlated with RL. Four *Glomus* OTUs were positively correlated with RLD but OTU 71 (*Paraglomus*) was negatively correlated with RLD. Eight *Claroideoglomus* and *Glomus* OTUs were positively correlated with SRL. However, 16 *Claroideoglomus* and *Glomus* OTUs and three *Glomus* OTUs were negatively correlated with RD and C:N ratio, respectively. Leaf N was positively correlated to five *Claroideoglomus* and *Glomus* OTUs and leaf C was positively correlated with seven *Glomus* and *Rhizophagus* OTUs but negatively correlated with OTU 43 (*Glomus*). Shoot biomass and R:S ratio were not correlated with the relative abundance of any OTU.

## Discussion

The success and benefits of commercial AMF can be determined by a number of factors such as species compatibility with the selected environment and soil properties [11, 61] and spatial competition with native AMF for root space and colonization [62]. In this study, except for product 1, all the commercial bioinoculants increased the percentage of mycorrhizal structures (arbuscules, vesicles and hyphae) compared to control plants (Fig. 1, Supp. Figure 3). Products 3, 4 and 5 resulted in the greater root length colonization (RLC), suggesting compatibility with the plant host and possible synergy with the AMF community colonizing the roots. Similar to our previous field study [14], product 1 did not significantly increase RLC compared to control plants. Interestingly, products 2, 3, and 4 all included the same nine AMF species, along with ectomycorrhizal fungi, bacterial species, and abiotic additives (except for product 2), yielding similar results in fungal structures. Similar to product 1, product 5 comprised four AMF species, but showed results more comparable to those of products 2, 3, and 4. Furthermore, products 2 and 5, absent of additional additives or microbes, exhibited performance similar to that of products 3 and 4. This suggest that fungal features are primarily driven by AMF species rather than additives. However other studies have observed the influence of additives [10, 14]. The observed variations in colonization may reflect the viability and abundance of AMF propagules [10], differences in colonization strategies (e.g., spores vs. root fragments) [10, 63], and/or presence and competition with the native AMF community [10, 14, 63], at least under the soil conditions of this study.

It is well established that AMF improve nutrient uptake of several essential nutrients in plants by rapidly responding to nutrient availability and expanding the soil exploration through their extraradical hyphal network [7, 16]. In agreement with our previous study [14] and other reports in leaves of grapevines [15, 16], we found that AMF bioinoculants improved nitrogen (N) concentration in leaves of treated plants, especially with products 3, 4 and 5, while product 1 did not generate a significant effect (Supp. Figure 4). However, products 1, 3 and 5 contained other organisms and added fertilizer that could influence N uptake. The lack of increase in leaf N by product 1 could potentially be attributed to inhibitory effects between the orchard soil microorganisms, the additives present in the specific bioinoculant and/or the orchard soil components [10]. Typically, the ratio of carbon to nitrogen (C: N) is a reliable indicator of plant growth rate and N utilization [66]. In this study the C: N ratio significantly decreased in treated plants, supporting the hypothesis that AMF inoculation improves leaf N accumulation and decreases the leaf C: N ratio [14, 65].

Two previous comprehensive meta-analyses [66, 67], emphasized that AMF often increased plant shoot, root, and total biomass, but decreased the ratio of root to shoot (R: S). Our results showed that AMF bioinoculants increased plant biomass (root, shoot and trunk), except for product 1 in shoot and trunk biomass and product 2 in shoot biomass (Supp. Figure 5). This biomass improvement, especially in root biomass, may have been promoted by the AMF, either via physiological changes of roots [69] or by a greater translocation of nutrients and water from the fungus to the plant [9]. Although not significantly different, the R: S ratio observed for all treatments may reflect that the roots of inoculated plants had to supply mineral nutrients and water to a relatively larger shoot [69].

The grapevine root system has been described as having large diameter fine roots, low root density and few root hairs [70]. Consequently, AMF play a key role in the vineyard system in increasing the surface area available for nutrient absorption [12–14]. However, limited information is available on the effects of AMF on grapevine root system morphology [14, 71]. It was hypothesized that plants with thinner roots and higher specific root length (SRL) have greater branching intensity and lower AMF colonization [72], whereas plants with thicker roots, lower SRL and less branching are more densely colonized by AMF [72, 73]. Both strategies allow plants to forage for multiple soil resources [68, 72]. Our findings align with previous studies in grapevine rootstocks [71] and *Vitis vinifera* cv. Riesling grafted onto rootstocks 3309C and SO4 [14], showing an increase in root length density (RLD) and SRL except for root diameter (RD), especially in plants inoculated with products 2, 3, 4 and 5 (Fig. 2). It is possible that Cabernet Sauvignon plants would benefit by adopting both strategies, i.e., smaller RD with higher SRL and AMF hyphae structure that could efficiently explore and take up nutrients and water from a greater soil volume. However, it is also possible that these root morphological changes may disappear when adequate nutrients are available.

The introduction of commercial AMF in soils with native AMF communities may disrupt local ecosystems, affecting native AMF and plant communities [74]. A notable example is the co-invasion of Pinaceae trees and their ectomycorrhizal symbionts in South America after introduction to prevent soil erosion [75]. Due to their mostly generalist host associations, AMF are not typically considered an invasion risk, but AMF bioinoculants can have negative [33, 76], positive [33, 77], neutral [78, 79] or mixed [74, 80] effects on the native AMF community colonizing the plant roots. These interactions can affect the abundance, structure and composition of native AMF communities as well as the plant performance. In our study, applying commercial AMF bioinoculants to grapevines roots growing in excavated, non-sterile orchard soil did not affect the relative abundance, composition, or structure of the native AMF community in the roots (Figs. 3, 5, 6), leading us to reject our hypothesis that inoculation would significantly alter the native AMF community in grapevine roots. Our data showed that most of the OTUs were shared by all treatments (Fig. 3), indicating similarities in the AMF community composition between inoculated and non-inoculated plants. In agreement with previous studies [78, 79] our findings support the assumption that introducing new and mixed commercial AMF strains to a native AMF community does not necessarily lead to competition or partial or total replacement of native AMF communities.

It is important to emphasize that our sequencing data, based on DNA, may have detected dormant or dead spores which could have contributed to the lack of treatment differences. However, we found that introducing commercial AMF strains altered grapevine root morphology, improved N content, and increased biomass. This suggests that bioinoculants may have interacted with the native AMF community, potentially affecting the function of native AMF and/or intraradical AMF colonization [78, 81]. Also, it is possible that inoculation did not impact the native AMF, likely because our bioinoculants contained widespread species of the Glomeraceae family, such as *Funneliformis mosseae, Rhizophagus clarus, and Rhizophagus intraradices,* which are commonly present in agricultural soils [62]. However, inoculation introduced new genotypes, which may have different functional traits than the native genotypes [81, 82], and may have adapted to local niche requirements, working synergistically with the native genotypes [79, 83]. Besides, our study was only six months in duration, hence the absence of an effect of commercial AMF on native AMF communities could reflect a lag time between the application of the bioinoculants and their effects on native AMF communities. Further long-term research is needed to determine the impact of AMF bioinoculants on the richness, diversity, and structure of native AMF communities that colonize grapevine roots.

Evaluating the effects of commercial AMF bioinoculants on native AMF is challenging because of the significant genetic polymorphism and functional diversity within AMF species [81]. The ITS, including ITS2 rRNA gene, is the standard fungal barcode [84], however, it is hypervariable within AMF and may underestimate Glomeromycotina diversity [84, 85]. To achieve better outcomes, the 18S rRNA gene has been commonly employed to analyze AMF communities [43, 85]. In this study both molecular markers, 18S and ITS2 rRNA genes, were employed. Unsurprisingly, the use of both molecular signatures is associated with challenges, including sequence variability, intragenomic variation, limited reference databases and lack of standardized procedures for taxonomic identification and validation [85]. Our study found that the 18S performed better than ITS2 in terms of recovery of a greater diversity of AMF (as expected), but both markers indicated no significant effect of treatment on the fungal community (Fig. 5). However, our current sequencing strategy did not allow us to discriminate and/or quantify the proportion of the introduced and the native AMF species colonizing the grapevine roots of this experiment. Further sequencing of the commercial inoculants using the same primer pairs employed for the sequencing of the roots would have enabled discrimination of the commercial AMF species from the native AMF species, but this was not included in our study.

The commercial bioinoculants evaluated in this study were expected to contain AMF species belonging to the Claroideoglomeraceae (*Claroideoglomus etunicatum*), Diversisporaceae (*Gigaspora margarita*), Glomeraceae (*Funneliformis monosporus*, *Funneliformis mosseae*, *Rhizophagus aggregatum*, *Rhizophagus clarus*, *Rhizophagus intraradices, Septoglomus deserticola*)*,* and Paraglomeraceae (*Paraglomus brasilianum*) families. The metabarcoding approach allowed us to establish that members of the Glomeraceae family were the most abundant AMF colonizing roots across all conditions, while Claroideoglomeraceae, Diversisporaceae, Paraglomeraceae, and *Septoglomus* were less abundant. At the species level we identified “potential species”, such as *Funneliformis mosseae*, and *Rhizophagus intraradices* (Fig. 6). These findings are consistent with previous studies conducted on different agricultural lands [6], including vineyards [86–88], which suggested that these species are regularly found in vine-growing areas worldwide [12, 87, 88]. According to the AMF life history strategies, being classified by Chagnon et al. (2013) [26] as competitors (C), stress tolerators (S) and ruderals (R) in the CSR framework, species belonging to Glomeraceae have short life cycles, are rapid colonizers with abundant production of spores, and have more efficient hyphal healing following disturbance [26, 64]. These phenotypic traits are characteristics of “ruderal” AMF species [26] and give species of the Glomeraceae family a competitive advantage in viticultural settings under conventional practices. In our study, we also identified OTUs from Claroideoglomeraceae and Diversisporaceae families (Supp. Figure 7), which is consistent with the results mentioned above. Additionally, we found taxa from the Paraglomeraceae family (Supp. Figure 7). This family was declared by the manufacturers to be present in products 2, 3 and 4. The presence of this family in vineyard soils and grapevine roots worldwide is still controversial. Some studies reported their absence, and other studies reported their presence and significantly colonized grapevine roots [88, 89].

We also noted a significant correlation between several *Claroideoglomus* and *Glomus* OTUs and root biomass, leaf N and C, and root morphological parameters (RL, RLD, SRL), while one OTU belonging to *Rhizophagus* was positively correlated to C (Fig. 7). The predominance of positive over negative correlations suggests a more synergistic interaction between introduced and native AMF communities, enhancing grapevine growth and performance [88]. Conversely, the relative abundance of 16 OTUs was negatively correlated with RD, suggesting that the root-associated AMF affects RD and complexity in the root system [14, 72, 90]. More molecular, metabolic and physiological research is needed to understand the interplay between plant root traits and AMF communities and their role in developing stress-resilient root systems that optimize nutrients, water uptake, and enhance soil carbon sequestration.

## Conclusion

This study demonstrates that commercial AMF bioinoculants applied to grapevine roots growing in a non-sterile orchard soil can successfully colonize roots and induce positive changes in grapevine root morphology, improving leaf N absorption and plant biomass, without necessarily altering the structure and composition of native AMF communities, suggesting a possible synergistic interaction to promote plant growth. These findings enhance our understanding of how bioinoculants interact with native AMF communities to affect grapevine performance and increase its resilience. However, further work to improve traceability of commercial bioinoculants containing a mix of AMF species is needed to distinguish between the introduced and the native AMF species colonizing grapevine roots. Moreover, future research should consider other types of inoculums (*i.e.*, native AMF isolates), multiple combinations of grapevine scion and rootstock, and the functional diversity within AMF species and their life history traits to better understand the relationships among these factors.

## Supplementary Information

Supplementary material 1

## Data Availability

All sequencing data for this study were uploaded to the NCBI Sequence Read Archive accession number PRJNA1136973. All other relevant data generated and analyzed during this study, are included in this article and its supplementary information file.

## Code availability

R scripts designed for data analyses and figure production are available at OSF (https://osf.io/kp65c/).

## References

[1] Ollat N, Cookson SJ, Destrac-Irvine A, Lauvergeat V, Ouaked-Lecourieux F, Marguerit E, et al. Grapevine adaptation to abiotic stress: an overview. Acta Hortic. 2019;1248:497–512. 10.17660/ActaHortic.2019.1248.68.

[2] Fichtl L, Hofmann M, Kahlen K, Voss-Fels KP, Saint Cast C, Ollat N, et al. Towards grapevine root architectural models to adapt viticulture to drought. Front Plant Sci. 2023;14:1162506. 10.3389/fpls.2023.1162506. 36998680 10.3389/fpls.2023.1162506 PMC10043487

[3] George NP, Ray JG. The inevitability of arbuscular mycorrhiza for sustainability in organic agriculture-A critical review. Front Sustain Food Syst. 2023;7:1124688. 10.3389/fsufs.2023.1124688.

[4] Del Buono D. Can biostimulants be used to mitigate the effect of anthropogenic climate change on agriculture? It is time to respond. Sci Total Environ. 2021;751:141763. 10.1016/j.scitotenv.2020.141763. 32889471 10.1016/j.scitotenv.2020.141763

[5] Brundrett MC, Tedersoo L. Evolutionary history of mycorrhizal symbioses and global host plant diversity. New Phytol. 2018;220:1108–15. 10.1111/nph.14976. 29355963 10.1111/nph.14976

[6] Stürmer SL, Bever JD, Morton JB. Biogeography of arbuscular mycorrhizal fungi (Glomeromycota): A phylogenetic perspective on species distribution patterns. Mycorrhiza. 2018;28:587–603. 10.1007/s00572-018-0864-6. 30187122 10.1007/s00572-018-0864-6

[7] Smith SE, Smith FA. Fresh perspectives on the roles of arbuscular mycorrhizal fungi in plant nutrition and growth. Mycologia. 2012;104:1–13. 10.3852/11-229. 21933929 10.3852/11-229

[8] Wahab A, Muhammad M, Munir A, Abdi G, Zaman W, Ayaz A, et al. Role of arbuscular mycorrhizal fungi in regulating growth, enhancing productivity, and potentially influencing ecosystems under abiotic and biotic stresses. Plants. 2023;12(17):3102. 10.3390/plants12173102. 37687353 10.3390/plants12173102 PMC10489935

[9] Begum N, Qin C, Ahanger MA, Raza S, Khan MI, Ashraf M, Ahmed N, Zhang L. Role of arbuscular mycorrhizal fungi in plant growth regulation: Implications in abiotic stress tolerance. Front Plant Sci. 2019;10:1068. 10.3389/fpls.2019.01068. 31608075 10.3389/fpls.2019.01068 PMC6761482

[10] Salomon MR, Demarmels SJ, Watts-Williams MJ, McLaughlin A, Kafle C, Ketelsen A, et al. Global evaluation of commercial arbuscular mycorrhizal inoculants under greenhouse and field conditions.". Appl Soil Ecol. 2022;169:104225. 10.1016/j.apsoil.2021.104225.

[11] Schütz L, Gattinger A, Meier M, Müller A, Boller T, Mäder P, et al. Improving crop yield and nutrient use efficiency via biofertilization-a global meta-analysis. Front Plant Sci. 2017;8:2204. 10.3389/fpls.2017.02204. 29375594 10.3389/fpls.2017.02204 PMC5770357

[12] Trouvelot S, Bonneau L, Redecker D, van Tuinen D, Adrian M, Wipf D. Arbuscular mycorrhiza symbiosis in viticulture: a review. Agron Sustain Dev. 2015;35:1449–67. 10.1007/s13593-015-0329-7.

[13] Torres N, Yu R, Kurtural K. Arbuscular mycorrhizal fungi inoculation and applied water amounts modulate the response of young grapevines to mild water stress in a hyper-arid season. Front Plant Sci. 2021;11:622209. 10.3389/fpls.2020.622209. 33519880 10.3389/fpls.2020.622209 PMC7840569

[14] Berdeja MP, Ye Q, Bauerle TL, Vanden Heuvel JE. Commercial bioinoculants increase root length colonization and improve petiole nutrient concentration of field grown grapevines. HortTechnology. 2023;33:48–58. 10.21273/HORTTECH05110-22.

[15] Schreiner RP. Effects of native and nonnative arbuscular mycorrhizal fungi on growth and nutrient uptake of ‘Pinot noir’ (*Vitis vinifera* L) in two soils with contrasting levels of phosphorus. Appl Soil Ecol. 2007;36:205–15. 10.1016/j.apsoil.2007.03.002.

[16] Nogales A, Santos ES, Abreu MM, Arán D, Victorino G, Pereira H, et al. Mycorrhizal inoculation differentially affects grapevine’s performance in copper contaminated and non-contaminated soils. Front Plant Sci. 2019;9:1906. 10.3389/fpls.2018.01906. 30740120 10.3389/fpls.2018.01906 PMC6355709

[17] Hao Z, Xie W, Chen B. Arbuscular mycorrhizal symbiosis affects alant immunity to viral infection and accumulation. Viruses. 2019;11:534. 10.3390/v11060534. 31181739 10.3390/v11060534 PMC6630321

[18] Nogales A, Aguirreolea J, Santa María E, Camprubí A, Calvet C. Response of mycorrhizal grapevine to *Armillaria mellea* inoculation: disease development and polyamines. Plant Soil. 2009;317:177–87. 10.1007/s11104-008-9799-6.

[19] Cruz-Silva A, Figueiredo A, Sebastiana M. First insights into the effect of mycorrhizae on the expression of pathogen effectors during the infection of grapevine with *Plasmopara viticola*. Sustainability. 2021;13:1226. 10.3390/su13031226.

[20] Vries JD, Evers JB, Kuyper TW, Ruijven JV, Mommer L. Mycorrhizal associations change root functionality: a 3D modelling study on competitive interactions between plants for light and nutrients. New Phytol. 2021;231(3):1171–82. 10.1111/nph.17435. 33930184 10.1111/nph.17435 PMC8361744

[21] Chen W, Ye T, Sun Q, Niu T, Zhang J. Arbuscular mycorrhizal fungus alters root system architecture in *Camellia sinensis* L as revealed by RNA-Seq analysis. Front Plant Sci. 2021;12:777357. 10.3389/fpls.2021.777357. 34868178 10.3389/fpls.2021.777357 PMC8636117

[22] Eissenstat DM, Kucharski JM, Zadworny M, Adams T, Koide RT. Linking root traits to nutrient foraging in arbuscular mycorrhizal trees in a temperate forest. New Phytol. 2015;208(1):114–24. 10.1111/nph.13451. 25970701 10.1111/nph.13451

[23] Antolín MC, Izurdiaga D, Urmeneta L, Pascual I, Irigoyen JJ, Goicochea N. Dissimilar responses of ancient grapevines recovered in Navarra (Spain) to arbuscular mycorrhizal symbiosis in terms of berry quality. Agronomy. 2020;10:473. 10.3390/agronomy10040473.

[24] Crossay T, Majorel C, Redecker D, Gensous S, Medevielle V, Durrieu G, et al. Is a mixture of arbuscular mycorrhizal fungi better for plant growth than single-species inoculants? Mycorrhiza. 2019;29:325–39. 10.1007/s00572-019-00898-y. 31203456 10.1007/s00572-019-00898-y

[25] Rosa D, Pogiatzis A, Bowen P, Kokkoris V, Richards A, Holland T, et al. Performance and establishment of a commercial mycorrhizal inoculant in viticulture. Agriculture. 2020;10:539. 10.3390/agriculture10110539.

[26] Chagnon PL, Bradley RL, Maherali H, Klironomos JN. A trait-based framework to understand life history of mycorrhizal fungi. Trends Plant Sci. 2013;18:484–91. 10.1016/j.tplants.2013.05.001. 23756036 10.1016/j.tplants.2013.05.001

[27] Grime JP. Evidence for the existence of three primary strategies in plants and its relevance to ecological and evolutionary theory. Am Nat. 1977;111:1169–94. 10.1086/283244.

[28] Marro N, Grilli G, Soteras F, Caccia M, Longo S, Cofré N, et al. The effects of arbuscular mycorrhizal fungal species and taxonomic groups on stressed and unstressed plants: a global meta-analysis. New Phytol. 2022;235(1):320–32. 10.1111/nph.18102. 35302658 10.1111/nph.18102

[29] Bettenfeld P, Cadena Canals J, Jacquens L, Fernandez O, Fontaine F, van Schaik E, et al. The microbiota of the grapevine holobiont: A key component of plant health. J Adv Res. 2022;40:1–15. 10.1016/j.jare.2021.12.008. 36100319 10.1016/j.jare.2021.12.008 PMC9481934

[30] Chaudhary VB, Holland EP, Charman-Anderson S, Guzman A, Bell-Dereske L, Cheeke TE, et al. What are mycorrhizal traits? Trends Ecol Evol. 2022;37(7):573–81. 10.1016/j.tree.2022.04.003. 35504748 10.1016/j.tree.2022.04.003

[31] Berruti A, Borriello R, Della Beffa MT, Scariot V, Bianciotto V. Application of nonspecific commercial AMF inocula results in poor mycorrhization in *Camellia japonica* L. Symbiosis. 2013;61:63–76. 10.1007/s13199-013-0258-7.

[32] Vahter T, Lillipuu EM, Oja J, Öpik M, Vasar M, Hiiesalu I. Do commercial arbuscular mycorrhizal inoculants contain the species that they claim? Mycorrhiza. 2023;33:211–20. 10.1007/s00572-023-01105-9. 36786883 10.1007/s00572-023-01105-9

[33] Basiru S, Hijri M. The potential applications of commercial arbuscular mycorrhizal fungal inoculants and their ecological consequences. Microorganisms. 2022;10(10):1897. 10.3390/microorganisms10101897. 36296173 10.3390/microorganisms10101897 PMC9609176

[34] Guo DL, Xia M, Wei X, Chang W, Liu Y, Wang Z. Anatomical traits associated with absorption and mycorrhizal colonization are linked to root branch order in twenty-three Chinese temperate tree species. New Phytol. 2008;180(3):673–83. 10.1111/j.1469-8137.2008.02573.x. 18657210 10.1111/j.1469-8137.2008.02573.x

[35] McCormack ML, Dickie IA, Eissenstat DM, Fahey TJ, Fernandez CW, Guo D, et al. Redefining fine roots improves understanding of belowground contributions to terrestrial biosphere processes. New Phytol. 2015;207(3):505–18. 10.1111/nph.13363. 25756288 10.1111/nph.13363

[36] Koske RE, Gemma JNA. Modified procedure for staining roots to detect VA mycorrhizas. Mycol Res. 1989;92:486–8. 10.1016/S0953-7562(89)80195-9.

[37] McGonigle TP, Miller MH, Evans DG, Fairchild GL, Swan JA. A new method which gives an objective measure of colonization of roots by vesicular-arbuscular mycorrhizal fungi. New Phytol. 1990;115(3):495–501. 10.1111/j.1469-8137.1990.tb00476.x. 33874272 10.1111/j.1469-8137.1990.tb00476.x

[38] Dumbrell AJ, Ashton D, Aziz N, Feng G, Nelson M, Dytham N, et al. Distinct seasonal assemblages of arbuscular mycorrhizal fungi revealed by massively parallel pyrosequencing. New Phytol. 2011;190(3):794–804. 10.1111/j.1469-8137.2010.03636.x. 21294738 10.1111/j.1469-8137.2010.03636.x

[39] Lee J, Lee S, Young JP. Improved PCR primers for the detection and identification of arbuscular mycorrhizal fungi. FEMS Microbiol Ecol. 2008;65(2):339–49. 10.1111/j.1574-6941.2008.00531.x. 18631176 10.1111/j.1574-6941.2008.00531.x

[40] Ihrmark K, Bödeker ITM, Cruz-Martinez K, Friberg H, Kubartova A, Schenck J, et al. New primers to amplify the fungal ITS2 region–evaluation by 454-sequencing of artificial and natural communities. FEMS Microbiol Ecol. 2012;82(3):666–77. 10.1111/j.1574-6941.2012.01437.x. 22738186 10.1111/j.1574-6941.2012.01437.x

[41] Kohout P, Sudová R, Janoušková M, Čtvrtlíková M, Hejda M, Pánková H, et al. Comparison of commonly used primer sets for evaluating arbuscular mycorrhizal fungal communities: Is there a universal solution? Soil Biol Biochem. 2014;68:482–93. 10.1016/j.soilbio.2013.08.027.

[42] White TJ, Bruns T, Lee S, Taylor JW. Amplification and direct sequencing of fungal ribosomal RNA genes for phylogenetics. In: Innis MA, Gelfand DH, Sninsky JJ, White TJ, editors. PCR Protocols: A guide to methods and applications. New York: Academic Press; 1990. p. 315–22.

[43] Lekberg Y, Vasar M, Bullington LS, Sepp SK, Antunes PM, Bunn R, et al. More bang for the buck? Can arbuscular mycorrhizal fungal communities be characterized adequately alongside other fungi using general fungal primers? New Phytol. 2018;220(4):971–6. 10.1111/nph.15035. 29388685 10.1111/nph.15035

[44] Palmer JM, Jusino MA, Banik MT, Lindner DL. Non-biological synthetic spike-in controls and the AMPtk software pipeline improve mycobiome data. PeerJ. 2018;6:e4925. 10.7717/peerj.4925. 29868296 10.7717/peerj.4925 PMC5978393

[45] Callahan BJ, McMurdie PJ, Rosen MJ, Han AW, Johnson AJA, Holmes SP. DADA2: High-resolution sample inference from Illumina amplicon data. Nat Methods. 2016;13:581–3. 10.1038/nmeth.3869. 27214047 10.1038/nmeth.3869 PMC4927377

[46] Rognes T, Flouri T, Nichols B, Quince C, Mahé F. VSEARCH: a versatile open source tool for metagenomics. PeerJ. 2016;4:e2584. 10.7717/peerj.2584. 27781170 10.7717/peerj.2584 PMC5075697

[47] Öpik M, Vanatoa A, Vanatoa E, Moora M, Davison J, Kalwij JM, et al. The online database MaarjAM reveals global and ecosystemic distribution patterns in arbuscular mycorrhizal fungi (Glomeromycota). New Phytol. 2010;188(1):223–41. 10.1111/j.1469-8137.2010.03334.x. 20561207 10.1111/j.1469-8137.2010.03334.x

[48] Edgar RC, Flyvbjerg H. Error filtering, pair assembly and error correction for next-generation sequencing reads. Bioinformatics. 2015;31:3476–82. 10.1093/bioinformatics/btv401. 26139637 10.1093/bioinformatics/btv401

[49] Abarenkov K, Henrik Nilsson R, Larsson KH, Taylor AFS, May TW, Guldberg Frøslev T, et al. The UNITE database for molecular identification and taxonomic communication of fungi and other eukaryotes: sequences, taxa and classifications reconsidered. Nucleic Acids Res. 2024;52:D791–7. 10.1093/nar/gkad1039. 37953409 10.1093/nar/gkad1039 PMC10767974

[50] R Core Team. R: A Language and Environment for Statistical Computing. R Foundation for Statistical Computing, Vienna, Austria. 2021

[51] McMurdie PJ, Holmes S. Phyloseq: An R package for reproducible interactive analysis and graphics of microbiome census data. PLoS ONE. 2013;8(4):e61217. 10.1371/journal.pone.0061217. 23630581 10.1371/journal.pone.0061217 PMC3632530

[52] Oksanen J, Simpson GL, Blanchet FG, Kindt R, Legendre P, Minchin PR, et al. vegan: Community Ecology Package. R package version 2.6–4. 2022. https://CRAN.R-project.org/package=vegan.

[53] Koleff P, Gaston KJ, Lennon JJ. Measuring beta diversity for presence-absence data. J Anim Ecol. 2003;72(3):367–82. 10.1046/j.1365-2656.2003.00710.x.

[54] Wickham H. ggplot2: Elegant graphics for data analysis. 2nd ed. Springer: Verlag; 2016.

[55] Anderson MJ. A new method for non-parametric multivariate analysis of variance. Austral Ecol. 2001;26:32–46. 10.1111/j.1442-9993.2001.01070.pp.x.

[56] Anderson MJ, Ellingsen KE, McArdle BH. Multivariate dispersion as a measure of beta diversity. Ecol Lett. 2006;9:683–93. 10.1111/j.1461-0248.2006.00926.x. 16706913 10.1111/j.1461-0248.2006.00926.x

[57] Krassowski M, Arts M, Lagger C. Krassowski/coplex-upset: v1.3.5 (v1.3.5). Zenodo. 2022. 10.5281/zenodo.7314197.

[58] Lex A, Gehlenborg N, Strobelt H, Vuillemot R, Pfister H. “UpSet: Visualization of intersecting sets. IEEE Trans Visual Comput Graphics. 2014;20(12):1983–92. 10.1109/TVCG.2014.2346248. 10.1109/TVCG.2014.2346248 PMC4720993 26356912

[59] Revelle W. psych: Procedures for Psychological, Psychometric, and Personality Research. Northwestern University, Evanston, Illinois. R package version 2.3.12. 2023. https://CRAN.R-project.org/package=psych.

[60] Kassambara A, Patil I. Package ‘ggcorrplot’: Visualization of a correlation matrix using 'ggplot2'. R package version 0.1.4.1. 2023. https://CRAN.R-project.org/package=ggcorrplot.

[61] García IV, Mendoza RE. Relationships among soil properties, plant nutrition and arbuscular mycorrhizal fungi–plant symbioses in a temperate grassland along hydrologic, saline and sodic gradients. FEMS Microbiol Ecol. 2008;63:359–71. 10.1111/j.1574-6941.2008.00441.x. 18205811 10.1111/j.1574-6941.2008.00441.x

[62] Verbruggen E, van der Heijden MG, Rillig MC, Kiers ET. Mycorrhizal fungal establishment in agricultural soils: factors determining inoculation success. New Phytol. 2012;197(4):1104–9. 10.1111/j.1469-8137.2012.04348.x. 10.1111/j.1469-8137.2012.04348.x 23495389

[63] Köhl L, Lukasiewicz CE, van der Heijden MG. Establishment and effectiveness of inoculated arbuscular mycorrhizal fungi in agricultural soils. Plant, Cell Environ. 2016;39(1):136–46. 10.1111/pce.12600. 26147222 10.1111/pce.12600

[64] Klironomos JN, Hart MM. Colonization of roots by arbuscular mycorrhizal fungi using different sources of inoculum. Mycorrhiza. 2002;12:181184. 10.1007/s00572-002-0169-6. 10.1007/s00572-002-0169-6 12189472

[65] Royer M, Larbat R, Le Bot J, Adamowicz S, Robin C. Is the C: N ratio a reliable indicator of C allocation to primary and defence-related metabolisms in tomato? Phytochemistry. 2013;88:25–33. 10.1016/j.phytochem.2012.12.003. 23312460 10.1016/j.phytochem.2012.12.003

[66] Veresoglou SD, Menexes G, Rillig MC. Do arbuscular mycorrhizal fungi affect the allometric partition of host plant biomass to shoots and roots? A meta-analysis of studies from 1990 to 2010. Mycorrhiza. 2012;22:227–35. 10.1007/s00572-011-0398-7. 21710352 10.1007/s00572-011-0398-7

[67] Qin M, Li L, Miranda JP, Tang Y, Song B, Oosthuizen MK, et al. Experimental duration determines the effect of arbuscular mycorrhizal fungi on plant biomass in pot experiments: A meta-analysis. Front Plant Sci. 2022;13:1024874. 10.3389/fpls.2022.1024874. 36407631 10.3389/fpls.2022.1024874 PMC9671359

[68] McCormack ML, Iversen CM. Physical and functional constraints on viable belowground acquisition strategies. Front Plant Sci. 2019;10:1215. 10.3389/fpls.2019.01215. 31681355 10.3389/fpls.2019.01215 PMC6797606

[69] Kothari SK, Marschner H, George E. Effect of VA mycorrhizal fungi and rhizosphere microorganisms on root and shoot morphology, growth and water relations in maize. New Phytol. 1990;116(2):303–11. 10.1111/j.1469-8137.1990.tb04718.x.

[70] Smart DV, Schwass E, Lakso A, Morano L. Grapevine rooting patterns: a comprehensive analysis and a review. Am J Enol Vitic. 2006;57:89–104. 10.5344/ajev.2006.57.1.89.

[71] Aguín O, Mansilla JP, Vilariño A, Sainz MJ. Effects of mycorrhizal inoculation on root morphology and nursery production of three grapevine rootstocks. Am J Enol Vitic. 2004;55:108–11. 10.5344/ajev.2004.55.1.108.

[72] Comas LH, Callahan HS, Midford PE. Patterns in root traits of woody species hosting arbuscular and ectomycorrhizas: implications for the evolution of belowground strategies. Ecol Evol. 2014;4:2979–90. 10.1002/ece3.1147. 25247056 10.1002/ece3.1147 PMC4161172

[73] Fitter AH. Magnolioid roots - hairs, architecture and mycorrhizal dependency. New Phytol. 2004;164(1):15–6. 10.1111/j.1469-8137.2004.01193.x. 33873490 10.1111/j.1469-8137.2004.01193.x

[74] Hart MM, Antunes PM, Chaudhary VB, Abbott LK, Field K. Fungal inoculants in the field: Is the reward greater than the risk? Funct Ecol. 2017;32:126–35. 10.1111/1365-2435.12976.

[75] Policelli N, Vietorisz C, Bhatnagar JM, Nuñez MA. Ectomycorrhizal fungi invasions in southern South America. In: Lugo MA, Pagano MC, editors. Fungal Biology. Cham: Springer; 2022. p. 2022.

[76] Berruti A, Lumini E, Bianciotto V. AMF components from a microbial inoculum fail to colonize roots and lack soil persistence in an arable maize field. Symbiosis. 2017;72:73–80. 10.1007/s13199-016-0442-7.

[77] Bender SF, Schlaeppi K, Held A, Van der Heijden MGA. Establishment success and crop growth effects of an arbuscular mycorrhizal fungus inoculated into Swiss corn fields. Agric Ecosyst Environ. 2019;273:13–24. 10.1016/j.agee.2018.12.003.

[78] Antunes PM, Koch AM, Dunfield KE, Hart MM, Downing A, Rillig MC, et al. Influence of commercial inoculation with Glomus intraradices on the structure and functioning of an AM fungal community from an agricultural site. Plant Soil. 2009;317:257–66. 10.1007/s11104-008-9806-y.

[79] Renaut S, Daoud R, Masse J, Vialle A, Hijri M. Inoculation with *Rhizophagus irregularis* does not alter arbuscular mycorrhizal fungal community structure within the roots of corn, wheat, and soybean crops. Microorganisms. 2020;8(1):83. 10.3390/microorganisms8010083. 31936180 10.3390/microorganisms8010083 PMC7023141

[80] Islam MN, Germida JJ, Walley FL. Survival of a commercial AM fungal inoculant and its impact on indigenous AM fungal communities in field soils. Appl Soil Ecol. 2021;166:103979. 10.1016/j.apsoil.2021.103979.

[81] Janoušková M, Krak K, Vosátka M, Püschel D, Štorchová H. Inoculation effects on root-colonizing arbuscular mycorrhizal fungal communities spread beyond directly inoculated plants. PLoS ONE. 2017;12(7):e0181525. 10.1371/journal.pone.0181525. 28738069 10.1371/journal.pone.0181525 PMC5524347

[82] Antunes PM, Koch AM, Morton JB, Rillig MC, Klironomos JN. Evidence for functional divergence in arbuscular mycorrhizal fungi from contrasting climatic origins. New Phytol. 2011;189(2):507–14. 10.1111/j.1469-8137.2010.03480.x. 20880038 10.1111/j.1469-8137.2010.03480.x

[83] Croll D, Giovannetti M, Koch AM, Sbrana C, Ehinger M, Lammers PJ, et al. Nonself vegetative fusion and genetic exchange in the arbuscular mycorrhizal fungus *Glomus intraradices*. New Phytol. 2009;181(4):924–37. 10.1111/j.1469-8137.2008.02726.x. 19140939 10.1111/j.1469-8137.2008.02726.x

[84] Tedersoo L, Bahram M, Zinger L, Nilsson RH, Kennedy PG, Yang T, et al. Best practices in metabarcoding of fungi: From experimental design to results. Mol Ecol. 2022;31:2769–95. 10.1111/mec.16460. 35395127 10.1111/mec.16460

[85] Öpik M, Zobel M, Cantero JJ, Davison J, Facelli JM, Hiiesalu I, et al. Global sampling of plant roots expands the described molecular diversity of arbuscular mycorrhizal fungi. Mycorrhiza. 2013;23:411–30. 10.1007/s00572-013-0482-2. 23422950 10.1007/s00572-013-0482-2

[86] Schreiner RP. Depth structures the community of arbuscular mycorrhizal fungi amplified from grapevine (*Vitis vinifera* L) roots. Mycorrhiza. 2020;30:149–60. 10.1007/s00572-020-00930-6. 31993741 10.1007/s00572-020-00930-6

[87] Cesaro P, Massa N, Bona E, Novello G, Todeschini V, Boatti L, et al. AMF communities in an Italian vineyard at two different phenological stages of *Vitis vinifera*. Front Microbiol. 2021;19:676610. 10.3389/fmicb.2021.676610. 10.3389/fmicb.2021.676610 PMC8326575 34349738

[88] Fors RO, Sorci-Uhmann E, Santos ES, Silva-Flores P, Abreu MM, Viegas W, et al. Influence of soil type, land use, and rootstock genotype on root-associated arbuscular mycorrhizal fungi communities and their impact on grapevine growth and nutrition. Agriculture. 2023;13:2163. 10.3390/agriculture13112163.

[89] Schreiner RP, Mihara KL. The diversity of arbuscular mycorrhizal fungi amplified from grapevine roots (*Vitis Vinifera* L) in Oregon vineyards is seasonally stable and influenced by soil and vine age. Mycologia. 2009;101(5):599–611. 19750939 10.3852/08-169

[90] Ramana JV, Tylianakis JM, Ridgway HJ, Dickie IA. Root diameter, host specificity and arbuscular mycorrhizal fungal community composition among native and exotic plant species. New phytol. 2023;239(1):301–10. 10.1111/nph.18911. 36967581 10.1111/nph.18911

