# Supplementary material for: Commercial bioinoculants improve colonization but do not alter the arbuscular mycorrhizal fungal community of greenhouse-grown grapevine roots
Source: Environ Microbiome. 2025 Jan 31;20:15. doi: 10.1186/s40793-025-00676-8 (PMC11783862; doi:10.1186/s40793-025-00676-8)
Supplement: Supplementary file 1 — Supplementary material 1 [file 40793_2025_676_MOESM1_ESM.docx]

**Supplementary Data**

**Supplementary Table 1.** Physicochemical parameters of the excavated and non-sterilized orchard soil applied in plants of *Vitis vinifera* cv. Cabernet Sauvignon, inoculated and non-inoculated (Control), with five bioinoculants containing arbuscular mycorrhizal fungi (AMF).

| **pH** | **Organic matter %** | **N**  **%** | **P**  **mg/kg** | **K**  **mg/kg** | **Ca**  **mg/kg** | **Mg**  **mg/kg** | **B**  **mg/kg** | **Zn**  **mg/kg** | **Cu**  **mg/kg** | **Mn**  **mg/kg** | **Fe**  **mg/kg** |  |
| --- | --- | --- | --- | --- | --- | --- | --- | --- | --- | --- | --- | --- |
| 6.54 | | 2.64 | 0.10 | 11.97 | 64.82 | 1571 | 342 | 0.45 | 0.49 | 0.31 | 12.53 | 4.18 |

**Supplementary Table 2**. Characteristics of commercial bioinoculants applied to roots of *Vitis vinifera* cv. Cabernet sauvignon.

| **Product no** | **AMF species** | **Other organisms**  **and additives** | **Propagule density ^1^ (grams) ^2^** |
| --- | --- | --- | --- |
| **1** | *Claroideoglomus etunicatum*  *Funneliformis mosseae*  *Rhizophagus aggregatum*  *Rhizophagus intraradices* | N, P, K  Humic acids  Softwood biochar  Worm castings | 20 propagules per gram for each species of the phylum Glomeromycota |
| **2** | *Claroideoglomus etunicatum*  *Funneliformis mosseae*  *Funneliformis monosporus*  *Gigaspora margarita*  *Paraglomus brasilianum*  *Rhizophagus clarus*  *Rhizophagus aggregatum*  *Rhizophagus intraradices*  *Septoglomus deserticola* | Clay | 50 propagules per gram for each species of the phylum *Glomeromycota* |
| **3** | *Claroideoglomus etunicatum*  *Funneliformis mosseae*  *Funneliformis monosporus*  *Gigaspora margarita*  *Paraglomus brasilianum*  *Rhizophagus clarus*  *Rhizophagus aggregatum*  *Rhizophagus intraradices*  *Septoglomus deserticola* | 7 Ectomycorrhizae  5 bacteria  1 Trichoderma  Kelp  Humic acids | 34 propagules per gram  for *R. aggregatum, R. irregularis, F. mosseae,* and *C. etunicatum*  13 propagules per gram for *F. monosporum, S. deserticola, R. clarum P. brasilianum* and *G. margarita* |
| 4 | *Claroideoglomus etunicatum*  *Funneliformis mosseae*  *Funneliformis monosporus*  *Gigaspora margarita*  *Paraglomus brasilianum*  *Rhizophagus clarus*  *Rhizophagus aggregatum*  *Rhizophagus intraradices*  *Septoglomus deserticola* | 7 Ectomycorrhizae  4 bacteria  N, P, K  Clay | 50 propagules per gram for each species of the phylum *Glomeromycota* |
| 5 | *Claroideoglomus etunicatum*  *Claroideoglomus claroideum*  *Funneliformis mosseae*  *Rhizophagus intraradices* | Clay | 50 propagules per gram for each species of the phylum *Glomeromycota* |

^1^ Manufacturer ’s information

^2^ One (1) propagule/g = 28.3495 propagules/oz

**
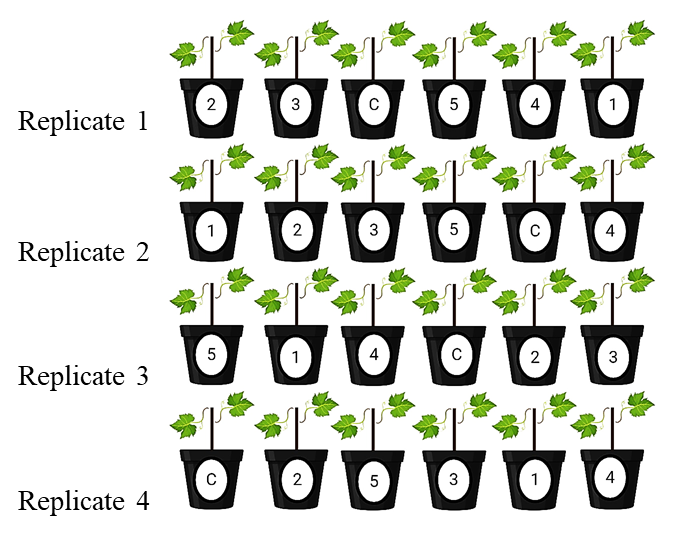
**

Experimental unit = 1 pot

Treatment = 6

Replications = 4

n Cabernet sauvignon/own rooted = 24 pots

C = Control (no bioinoculant applied).

See Additional Table 2 for description of treatments

**Supplementary Fig. 1.** Experimental setup of *Vitis vinifera* cv. Cabernet Sauvignon, inoculated and non-inoculated (Control), with five bioinoculants, growing in excavated orchard soil under greenhouse conditions.

**Supplementary Fig. 2.** Rarefaction curves for 18S rRNA gene region.

**Supplementary Fig. 3.** Percentage of arbuscular mycorrhizal fungal structures; arbuscules (A), vesicles (B) and hyphae (C) colonizing fine roots of *Vitis vinifera* cv. Cabernet Sauvignon (n= 4), inoculated and non-inoculated (Control), with five bioinoculants, growing in excavated orchard soil under greenhouse conditions. The boxplots show the first and third quartile ranges, with the line in the box representing the median. The whiskers extend from the first and third quartiles to values that are not within 1.5 x interquartile range from both directions. Data beyond the whiskers are presented as individual circles. Letters indicate differences in AMF colonization among inoculum treatments detected using Tukey’s honest significant difference post-hoc test derived from the linear model analysis of variance at α = 0.05.

**Supplementary l Fig. 4.** Leaf nitrogen (A), and carbon (B) concentration, and carbon to nitrogen ratio (C) and root length (mm) of *Vitis vinifera* cv. Cabernet Sauvignon (n= 4), inoculated and non-inoculated (Control), with five bioinoculants, growing in excavated orchard soil under greenhouse conditions. The boxplots show the first and third quartile ranges, with the line in the box representing the median. The whiskers extend from the first and third quartiles to values that are not within 1.5 x interquartile range from both directions. Data beyond the whiskers are presented as individual circles. Letters indicate differences in AMF colonization among inoculum treatments detected using Tukey’s honest significant difference post-hoc test derived from the linear model analysis of variance at α = 0.05.

**Supplementary Fig. 5.** Shoot (A), trunk (B) and root (C) dry biomass and root to shot ratio (D) of *Vitis vinifera* cv. Cabernet Sauvignon (n= 4), inoculated and non-inoculated (Control), with five bioinoculants, growing in excavated orchard soil under greenhouse conditions. The boxplots show the first and third quartile ranges, with the line in the box representing the median. The whiskers extend from the first and third quartiles to values that are not within 1.5 x interquartile range from both directions. Data beyond the whiskers are presented as individual circles. Letters indicate differences in AMF colonization among inoculum treatments detected using Tukey’s honest significant difference post-hoc test derived from the linear model analysis of variance at α = 0.05.

**Supplementary Fig. 6.** Taxonomic composition of fungal community composition as detected by the ITS2 gene region associated to roots of *Vitis vinifera* cv. Cabernet Sauvignon inoculated and non-inoculated (Control) with five bioinoculants, growing in excavated orchard soil under greenhouse conditions. Stacked bars represent relative abundance percentage and are colored by class level.

**Supplementary Fig. 7.** Taxonomic composition of AMF community operational taxonomic units (OTUs) for 18S (**A**) and ITS2 (**B**) rRNA gene regions, associated with roots of *Vitis vinifera* cv. Cabernet Sauvignon, inoculated and non-inoculated (Control) with five bioinoculants. Underlined species represent similar OTUs detected based on both gene regions. *Glomus* Wirsel and *Glomus* PorrasAlfaro are based off the MaarjAM virtual taxa (VT) sequences, included in the reference 18S database installed in the AMPtk pipeline. Stacked bars represent relative abundance (percentage) and are colored by species identification.

**Supplementary Table 3**. Comparison of arbuscular mycorrhizal fungi (18S region) and fungal (ITS region) community structure dissimilarity using permutational multivariate analysis of variance (PERMANOVA) for roots of *Vitis vinifera* cv. Cabernet Sauvignon, inoculated or not with five bioinoculants, growing in excavated orchard soil under greenhouse conditions. The p-values of dispersion test were derived from ANOVA.

| **BETA DISPERSION** | **Factor** | **Df** | **SS** | **MSS** | **F** | **Pr (>F)** |
| --- | --- | --- | --- | --- | --- | --- |
| Root 18S | Treatment | 5 | 0.011993 | 0.002398 | 3.0339 | 0.03697* |
|  | Residual | 18 | 0.014231 | 0.000790 |  |  |
| Root ITS | Treatment | 5 | 0.008638 | 0.001727 | 2.5948 | 0.0617 |
|  | Residual | 18 | 0.011985 | 0.000665 |  |  |
| **PERMANOVA** | **Factor** | **Df** | **SS** | **R2** | **F** | **P** |
| Root 18S | Treatment | 5 | 0.15233 | 0.21723 | 0.999 | 0.4824 |
|  | Residual | 18 | 0.54891 | 0.78277 |  |  |
|  | Total | 23 | 0.70123 | 1.00000 |  |  |
| Root ITS | Treatment | 5 | 0.33975 | 0.20107 | 0.906 | 0.7923 |
|  | Residual | 18 | 1.34996 | 0.79893 |  |  |
|  | Total | 23 | 1.68971 | 1.00000 |  |  |

Df: Degrees of freedom; SS: Sum of squares; R2: squared or coefficient of determination; F: F-statistics; P: P-value for F-statistics.
